# Protecting patients by training physicians in fluoroscopic radiation management

**DOI:** 10.1120/jacmp.v1i1.2653

**Published:** 2000-01-01

**Authors:** Benjamin R. Archer, Louis K. Wagner

**Affiliations:** ^1^ Department of Radiology Baylor College of Medicine Houston Texas 77030; ^2^ Department of Radiology The University of Texas‐Houston Medical School Houston Texas 77030

**Keywords:** radiation burns, fluoroscopy, radiation management

## Abstract

During the past 15 years, developments in *x*‐ray technologies have substantially enhanced the ability of practitioners to treat patients using fluoroscopically guided interventional techniques. However, many of these procedures require a greater use of fluoroscopy and serial imaging (cine). This has increased the potential for radiation‐induced dermatitis, epilation, and severe radiation‐induced burns to patients. It has also increased the potential for radiation injury and radiation‐induced cancer in personnel. This work will describe a number of the cases that have appeared in the literature and current recommendations and credentialing requirements of various organizations whose members use fluoroscopy. Finally, a program for implementing training of physicians in radiation management as a means of reducing the risk of injury to patients and personnel is recommended.

PACS number(s): 87.52.–g, 87.90.+y

## INTRODUCTION

**Figure 1:**
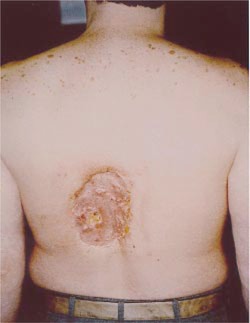
(Color) Radiation wound 22 months after angioplasty procedure.^2^

In 1994, the Center for Devices and Radiological Health of the United States Food and Drug Administration (FDA) issued an advisory^1^ warning healthcare facilities of the potential for radiation‐induced burns to patients from fluoroscopic procedures. A separate article cited the growing number of cases of severe injury.^2^ To date, the FDA has documented some 50 cases of radiation‐induced burns. European investigations have confirmed at least 15 cases of radiation dermatitis that resulted from cardiologic procedures.^3^ ^–^ ^7^ Additional case histories of injuries to both patients and physicians^8^ ^–^ ^11^ have appeared in the literature. Some of the radiation‐induced wounds discussed in these studies have required skin grafts resulting in permanent disfigurement. Cataracts and serious radiation injuries to hands have also been observed in physicians who have recently (as late as 1994) started using fluoroscopy in their practice (see Fig. 1).^12^ ^,^ ^13^

The FDA advisory alerted facilities to assure proper training of fluoroscopy personnel in light of “occasional but severe” radiation injuries from invasive procedures. Specific recommendations of this Advisory include the following:

(i) that all operators be trained and understand system operation, including the implications for radiation exposure from each mode of operation;
(ii) that facilities ensure that physicians performing fluoroscopic procedures have education so they may, on a case‐by‐case basis, assess risks and benefits for individual patients, considering variables such as age, beam location and direction, tissues in the beam and previous fluoroscopic procedures or radiation therapy;
(iii) that patients be counseled regarding the symptoms and risks when radiation exposures are expected to be high;^14^
(iv) that physicians justify and limit the use of high dose rate modes of operation;
(v) that facilities assure appropriate credentials and training for physicians performing fluoroscopy.

## CREDENTIALING PROCESS

The credentialing process for healthcare providers was originally instituted to protect patients from unethical or untrained practitioners by recognizing the competence of a professional.^15^ Recognition of competence may be through certification, licensure, registration, or a combination of the three. It nearly always involves the completion of an accredited or approved educational program. The credentialing of a medical staff member implies the verification and assessment of the practitioner's qualifications and the granting and delineation of clinical privileges. There is a distinction between the credentialing of a cardiologist, radiologist, pain interventionalist, or other fluoroscopist as technically competent to perform a procedure versus the credentialing of the same physician as competent to safely use fluoroscopic radiation. The Accreditation Council for Graduate Medical Education (ACGME) establishes Institutional and Program Requirements to insure that resident physicians are appropriately trained to perform clinical procedures with technical proficiency. The program requirements “specify essential educational content, instructional activities, responsibilities for patient care and supervision, and the necessary facilities of accredited programs in a particular specialty.” However, the current program requirements of the ACGME for most specialties that use fluoroscopy do not specifically mandate that resident physicians learn about radiation management in fluoroscopic procedures. Therefore, credentialing these physicians as technically competent gives no assurances that they have received training in the safe uses of fluoroscopy. Untrained practitioners logically should not be granted “rubber stamp” privileges^17^ to perform fluoroscopic procedures. The ACGME radiology residency program and the certification examination of the American Board of Radiology require technical proficiency plus comprehensive knowledge of the physics of medical imaging, the biological effects of radiation, and radiation safety. Therefore, completion of a residency and board certification in radiology implies formal education in the safe use of fluoroscopy. It does not, however, assure training in the safe applications for specific high‐risk procedures.

## POSITIONS OF ORGANIZATIONS ON EDUCATION OF FLUOROSCOPISTS

Many prominent cardiology organizations strongly advocate educational initiatives. The American College of Cardiology Consensus Document, “Radiation Safety in the Practice of Cardiology”^18^ clearly delineates radiation safety for cardiology staff. The summary statement defines the exigent need for training of cardiologists and support personnel:

“Given the large numbers of cardiac procedures involving radiation being performed in the United States by an increasing number of workers, the principles for reducing radiation and monitoring exposure should be known and followed by every practitioner, trainee, and assistant in every laboratory.”

Additionally, the report states that:

“It is strongly recommended that formal didactic sessions be incorporated into the training of physicians and other medical personnel who work in catheterization, electrophysiology and nuclear laboratories. The content should include basic radiation physics, radiation biology, radiation safety practices, monitoring procedures and potential health risks. Training sessions should be completed before beginning any participation in cardiology laboratories and annually thereafter.”

An American Heart Association—Medical/Scientific Statement—Special Report “Optimal Resources for the Examination and Endovascular Treatment of the Peripheral and Visceral Vascular Systems”^19^ was published in 1994. A task force that included members from the Councils on Cardiovascular Radiology, Cardio‐Thoracic and Vascular Surgery, Clinical Cardiology, and Kidney in Cardiovascular Disease authored the document. The report categorically states that “all angiographic and interventional physicians must have documented training in radiation physics, radiation biology and radiation safety.”

In 1995, the ACC Cardiac Catheterization Committee published a Position Statement^20^ that further indicates that appropriate training is imperative:

“Proper instruction in the principals of radiation physics and safety should be a part of every cardiologist's education. Unfortunately, this aspect of fellowship training often receives low priority even among physicians intending to base their careers in the cathetherization laboratory. Furthermore, knowledge gained in this area is not assessed by the specialty board examination.”

The recommendations of these groups are well justified and much needed but to date little progress has been made in implementing them.

We stress that training must include pertinent aspects of radiation management in the clinical setting in order that physicians know how to maintain risks to patients and personnel at acceptable levels. Training for interventional work should be procedure specific while incorporating general principles of safe practice. Requiring generalized physics training alone is insufficient (and often irrelevant) to assure appropriate radiation management. This is apparent from the numerous cases of burns from transjugular intrahepatic portosystemic shunt (TIPS) procedures, only some of which are reported in the literature^8^ (we know of others). These injuries are frequently associated with difficult, protracted procedures in large patients. Although radiologists frequently have physics training, they are not necessarily trained in effective radiation dose management in these long and difficult procedures.

Pain management using fluoroscopically guided procedures has proliferated in recent years. Physicians involved in this type of work should also be trained in the safe use of fluoroscopic radiation. Our experience is that training in this area is also lacking. One of the authors (L.K.W.) has personally observed at least two physicians who have radiation damage to their hands from placing them too frequently in the beam. Observed changes include discolored fingernails, epidermal degeneration, atrophy, and reduced healing capability from minor cuts and injury.

## A CASE STUDY

A middle‐aged woman had a history of progressively worsening episodes of arrhythmia. A radiofrequency electrophysiological cardiac catheter ablation was scheduled to treat the condition. The procedure employed 20 min of beam‐on time for each plane of a biplane fluoroscope.

**Figure 2:**
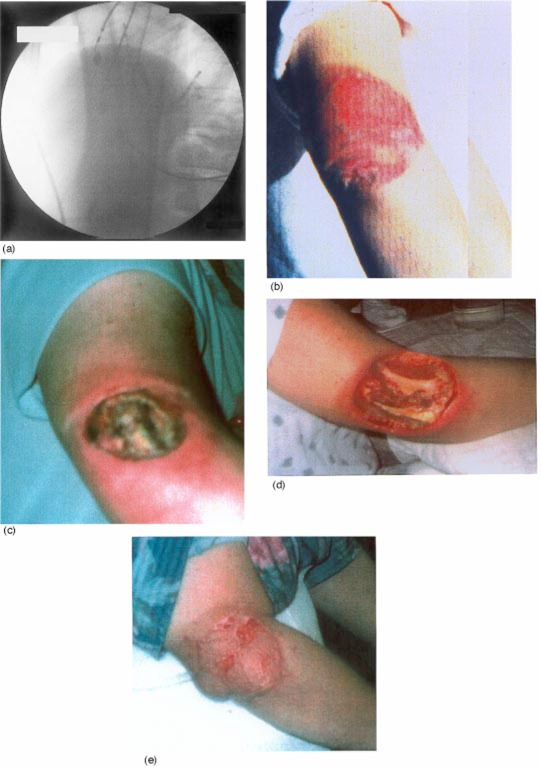
(Color) Progression of a wound in a patient following cardiac ablation procedure. The arm remained in the primary beam near the port of the *x*‐ray tube with the separator cone removed. (a) Fluorographic image illustrating arm in the beam; (b) erythema about 3 weeks after procedure; (c) ulcer at 5 months after procedure; (d) debridement at 8 months after the procedure; (e) surgical flap 10 months after procedure. (Reproduced with permission from Ref. 21.)

Prior to the procedure the separator cones were removed so that the fluoroscopic c‐arms could be easily rotated around the patient. The separator cone is a spacer attached to the tube housing designed to keep the patient at a reasonable distance from the *x*‐ray source. This is done specifically to avoid the high skin‐dose rates that can be encountered near the tube port. The right‐side fluoroscope was in a left‐anterior‐oblique orientation. The patient's arms were originally placed at the patient's side but the right arm later fell into a lower position directly in front of this *x*‐ray tube. However, personnel were not aware of this change because sterile covers were draped over the patient. The right humerus was directly in the beam at the port [see Fig. 2(a)].

Because the separator cones were removed, the arm was only about 20–30 cm from the focal spot. With the soft tissue and bone of the arm directly in the beam, the automatic brightness control drove the output to high levels at the surface of the arm. The machine was a high‐dose‐rate unit. In the normal mode of operation the output at the skin of the arm would have exceeded 500 mGy per minute due to the inverse‐square law and the close proximity of the skin to the source. In the high‐dose‐rate mode, the skin dose rate could have exceeded 1.5 Gy per minute. The cumulative dose probably exceeded 25 Gy.

The patient was released from the hospital the day after the procedure. At the time there were no complaints regarding her arm and no indication of erythema. About three weeks after the procedure, a bright erythema was demonstrated [Fig. 2(b)]. The condition worsened and at five months a large ulcer the size of the collimated *x*‐ray port developed [Fig. 2(c)]. At eight months a debridement was performed and a surgical flap was put in place [Figs. 2(d) and 2(e)].

## RECOMMENDATIONS

It is likely that many of the injuries to patients and staff reported in the literature and elsewhere could have been avoided or reduced in severity if physicians were trained in radiation management specifically for the procedures they perform. This will require new educational initiatives to ensure that procedure‐specific dose‐reduction techniques are taught to practioners in each specialty. Several radiation management issues are of note from this case study:

(1) the separator cone was removed;
(2) the arm was directly in the field;
(3) the port of the *x*‐ray collimators was nearly in contact with the arm.

To minimize the intensity of the radiation incident on the patient, the *x*‐ray source should always be moved as far away as possible from the patient's skin. This is especially important when the separator cone is removed. Body parts that need not be in the beam should be moved out of the field to avoid their unnecessary irradiation and to lower the radiation output required by the automatic brightness system. Further dose reductions to patient and personnel can be achieved by use of appropriate collimation, pulsed fluoroscopy and the use of magnification only when necessary. Our experience is that physicians sometimes focus on their own protection while failing to realize that radiation management for the patient they help safeguard all involved. Training on fundamental points such as these is essential for the avoidance of such injuries.

The recommendations of the authoritative documents discussed above and the slow but continuous stream of serious injuries will hopefully galvanize the American College of Cardiology and other organizations whose practioners use fluoroscopy to follow guidelines similar to those established by the ACGME and ABR for radiology. Training in radiation management and testing of every physician resident and/or fellow whose practice involves the use of fluoroscopy must be required. A template for some change has already been established. In 1999, the American Board of Internal Medicine conducted the inaugural certification examination in Interventional Cardiology. Ten percent of this examination covers imaging, which includes radiation physics and safety topics. This is an important *first* step for cardiology. Pain management, Orthopedics, and Urology programs should begin the process of incorporating formal training for all physicians involved in these procedures Radiology can improve their training by focussing on radiation management in specific areas and making procedure‐specific training a part of every program.

## ACKNOWLEDGMENTS

The authors wish to thank Drs. Jeffrey A. Brinker and John F. Cardella for their constructive comments regarding this manuscript.
